# Reference values of renal tubular function tests are dependent on age and kidney function

**DOI:** 10.14814/phy2.13542

**Published:** 2017-12-07

**Authors:** Anneke P. Bech, Jack F.M. Wetzels, Tom Nijenhuis

**Affiliations:** ^1^ Department of Nephrology Radboud University Medical Center Nijmegen The Netherlands

**Keywords:** DDAVP test, furosemide fludrocortisone test, furosemide test, reference value, thiazide test, tubular function test

## Abstract

Electrolyte disorders due to tubular disorders are rare, and knowledge about validated clinical diagnostic tools such as tubular function tests is sparse. Reference values for tubular function tests are based on studies with small sample size in young healthy volunteers. Patients with tubular disorders, however, frequently are older and can have a compromised renal function. We therefore evaluated four tubular function tests in individuals with different ages and renal function. We performed furosemide, thiazide, furosemide‐fludrocortisone, and desmopressin tests in healthy individuals aged 18–50 years, healthy individuals aged more than 50 years and individuals with compromised renal function. For each tubular function test we included 10 individuals per group. The responses in young healthy individuals were in line with previously reported values in literature. The maximal increase in fractional chloride excretion after furosemide was below the lower limit of young healthy individuals in 5/10 older subjects and in 2/10 patients with compromised renal function. The maximal increase in fractional chloride excretion after thiazide was below the lower limit of young healthy individuals in 6/10 older subjects and in 7/10 patients with compromised renal function. Median maximal urine osmolality after desmopressin was 1002 mosmol/kg H_2_O in young healthy individuals, 820 mosmol/kg H_2_O in older subjects and 624 mosmol/kg H_2_O in patients with compromised renal function. Reference values for tubular function tests obtained in young healthy adults thus cannot simply be extrapolated to older patients or patients with compromised kidney function. Larger validation studies are needed to define true reference values in these patient categories.

## Introduction

Electrolyte disorders can be the consequence of acquired or inherited renal tubular defects that cause malfunctioning of channels and transporters that are critically involved in the (re)absorption or secretion of electrolytes. These tubular disorders are rare, and knowledge about as well as experience with validated clinical diagnostic tools such as tubular function tests is sparse. Tubular function tests are valuable because they can help to elucidate the underlying defect(s) and can direct more specific diagnostic tests and therapy. Reference values for tubular function tests are mainly based on studies with small sample size in young healthy volunteers (Nozu et al. 2010; Miller et al. 1970; Colussi et al. 2007; Walsh et al. 2007). Patients with tubular disorders, however, are frequently older and/or have a compromised renal function. It is important to know whether we can use the available reference values when using these tubular function tests in such patients.

The aim of this study was to evaluate four different tubular function tests in our institution in young healthy individuals, older individuals and patients with compromised renal function (CRF). We performed the furosemide test that provides information on transcellular sodium‐chloride reabsorption in the thick ascending limb of Henle (typically used in the diagnosis of presumed Bartter syndrome), the thiazide test that reflects activity of sodium‐chloride reabsorption in the distal convoluted tubule (typically used in the diagnosis of presumed Gitelman syndrome), the furosemide‐ fludrocortisone (FF) test that can be used to diagnose distal renal tubular acidosis and the DDAVP test which assesses urinary concentration upon stimulating the collecting ducts with synthetic desmopressin (DDAVP) used in the diagnosis of a renal concentration defect and/or nephrogenic diabetes insipidus.

## Materials and Methods

### Study population

We performed four different tubular function tests in three groups of volunteers: healthy individuals aged 18–50 years, healthy individuals over 50 years old and patients with CRF. CRF was defined as an eGFR of less than 90 mL/min/1.73 m^2^ based on the CKD‐EPI formula. For each tubular function test, we included 10 individuals per group. Exclusion criteria were pregnancy, severe heart failure, disorders of sodium or potassium balance, kidney disease primarily involving the kidney tubule such as tubulo‐interstitial nephritis or suggested by an urinary alpha‐1‐microglobulin level of ≥40 mg/10 mmol creatinine. After informed consent, each volunteer participated in one or more tubular function tests. The tests were performed in the Radboud university medical center by a group of trained nurses. If participating in more than one test, the tests were performed at least 7 days apart. This study was approved by the medical ethics committee of the Radboud university medical center in Nijmegen, the Netherlands, and all participants gave written informed consent.

Duplicate urine and serum samples of all tests were stored in a freezer at −80°C.

To investigate whether there were differences between the use of an oral fluid load or an intravenous fluid load during the furosemide test and thiazide test, both tests were performed in duplicate in five young healthy volunteers: once with an oral fluid load as described below and once using NaCl 0.45% at 250 mL/h during the test. No statistically significant differences between oral and intravenous fluid loads were noted with respect to furosemide and thiazide test results (data not shown). We therefore choose to perform furosemide and thiazide tests with an oral fluid load.

The protocols of the tubular function tests are based on previous reports in literature (Nozu et al. 2010; Miller et al. 1970; Colussi et al. 2007; Walsh et al. 2007).

### Furosemide test

In the 7 days preceding the test, subjects were not allowed to take diuretics or non steroidal anti‐inflammatory drugs (NSAID's). In the 3 days preceding the test, subjects were not allowed to take angiotensin converting enzyme inhibitors (ACEi) or angiotensin receptor blockers (ARB). On the morning of the test, subjects were allowed to have a small breakfast without coffee. During the test, participants were allowed to eat one sandwich. They visited the clinic at 8:00 h. At that time (*T* = 0), body weight and blood pressure were measured and a urine sample was taken. Thereafter, they were instructed to drink 10 mL/kg of body weight water in 15 min time. After 45, 90, and 120 min, a urine sample was collected. After 150 min (*T* = 150), blood and urine samples were collected and one dose of furosemide was given orally. If the eGFR was >60 mL/min/1,73 m^2^, 40 mg of furosemide was given. If the eGFR was between 30 and 60 ml/min/1.73 m^2^, 60 mg furosemide was given, and if the eGFR was below 30 ml/min/1.73 m^2^, 80 mg of furosemide was given. From this moment on, the subject was instructed to drink 250 mL of water per hour until the end of the test. Urine was collected every 30 min until *T* = 330 min and at *T* = 270 an additional blood sample was collected. At the end of the test (*T* = 330), body weight and blood pressure were measured again.

### Thiazide test

In the 7 days preceding the test, subjects were not allowed to take diuretics or NSAID's. In the 3 days preceding the test, subjects were not allowed to take ACEi or ARB. On the morning of the test, subjects were allowed to have a small breakfast without coffee. During the test, participants were allowed to eat one sandwich. They visited the clinic at 8:00 h. At that time (*T* = 0), body weight and blood pressure were measured and a urine sample was taken. Thereafter, they were instructed to drink 10 mL/kg of body weight water in 15 min time. After 45, 90, and 120 min, a urine sample was collected. After 150 min (*T* = 150), blood and urine samples were collected and 50 mg of hydrochlorothiazide was given orally. From this moment on, the subject was instructed to drink 250 mL of water per hour until the end of the test. Urine was collected every 30 min until *T* = 510 min and at *T* = 270 and *T* = 510, an additional blood sample was collected. At the end of the test (*T* = 510), body weight and blood pressure were measured again.

### Furosemide Fludrocortisone test (FF test)

In the 7 days preceding the test, subjects were not allowed to take diuretics, NSAID's or sodium bicarbonate. On the morning of the test, the subjects were allowed to have a small breakfast without coffee. Subjects visited the clinic at 8:00 h in the morning. At that time, body weight and blood pressure were measured, blood was withdrawn, a urine sample was taken and 40 mg of furosemide and 1 mg of fludrocortisone were given orally. If the eGFR was between 30 and 60 mL/min/1.73 m^2^, a gift of 60 mg furosemide was given. If the eGFR was below 30 mL/min/1.73 m^2^, a gift of 80 mg of furosemide was given. Thereafter, urine samples were collected every hour during four hours. After 120 min (*T* = 120) and 240 min (*T* = 240) an additional blood sample was withdrawn. At the end of the test (*T* = 240), body weight and blood pressure were measured again.

### DDAVP test

In the 7 days preceding the test, subjects were not allowed to take diuretics or NSAID's. In the 24 h preceding the test, the subjects were not allowed to smoke cigarettes or drink alcohol or coffee. From 22:00 h on the evening before the test, they were not allowed to drink anymore, and during the test they were not allowed to drink or eat. The subjects visited the clinic at 8:00 h in the morning. At that time (*T* = 0) body weight and blood pressure were measured, blood was withdrawn, a urine sample was taken and 4 *μ*g of desmopressin was given subcutaneously. Thereafter, urine was collected every hour during 6 h. At *T* = 180 and *T* = 360 an additional blood sample was withdrawn. At the end of the test (*T* = 360), body weight and blood pressure were measured again.

### Calculations and assays

Urinary chloride was measured using an ion selective electrode for the cobas system (Roche Diagnostics). If the urine chloride concentration was reported as <20 mmol/L, calculations were done with 20 mmol/L. Fractional chloride excretion (FeCl) was calculated using the formula ((urine chloride * serum creatinine)/(urine creatinine * serum chloride)) *100. Urinary concentration of hydrochlorothiazide was measured by liquid chromatography‐mass spectrometry (LCMS). Urine pH was measured using a pH meter (PHM220, Hach, Tiel, the Netherlands).

### Statistics

Baseline characteristics are reported as median values with interquartile ranges. For statistical analyses, results in the experimental groups (either the elderly or the patients with CRF) were compared with results in the young healthy controls. Differences were analyzed with the Mann–Whitney U test. Differences between groups for categorical values were analyzed by the chi square test. A result was considered statistically significant if the *P* value was <0.03 according to the Bonferroni correction.

## Results

**Figure 1:**
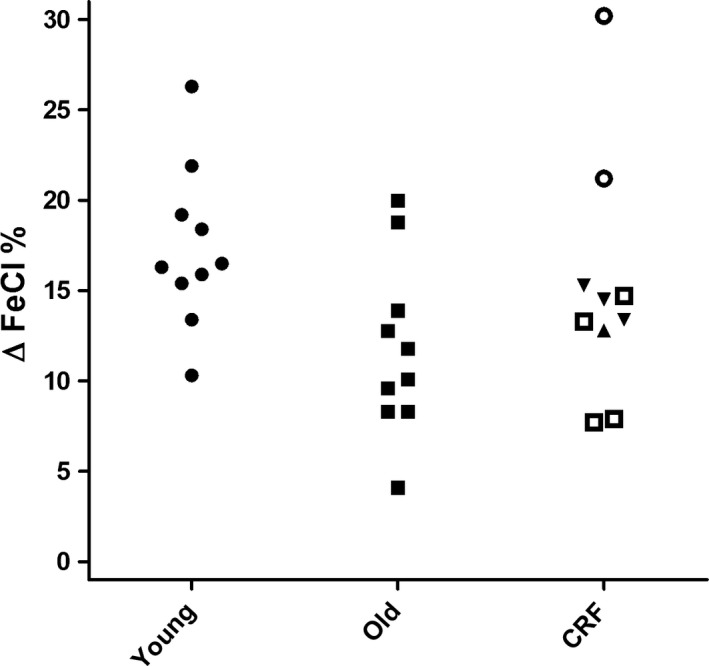
Furosemide test. Maximal increase in fractional excretion of chloride (∆FeCl) after administration of furosemide in young healthy individuals (young), older healthy individuals (old), and patients with compromised renal function (eGFR 60–89 mL/min/1.73 m^2^ □, eGFR 45–60 mL/min/1.73 m^2^ ▼, eGFR 30–44 mL/min/1.73 m^2^ ○ and eGFR 15–29 mL/min/1.73 m^2^ ▲).

**Figure 2:**
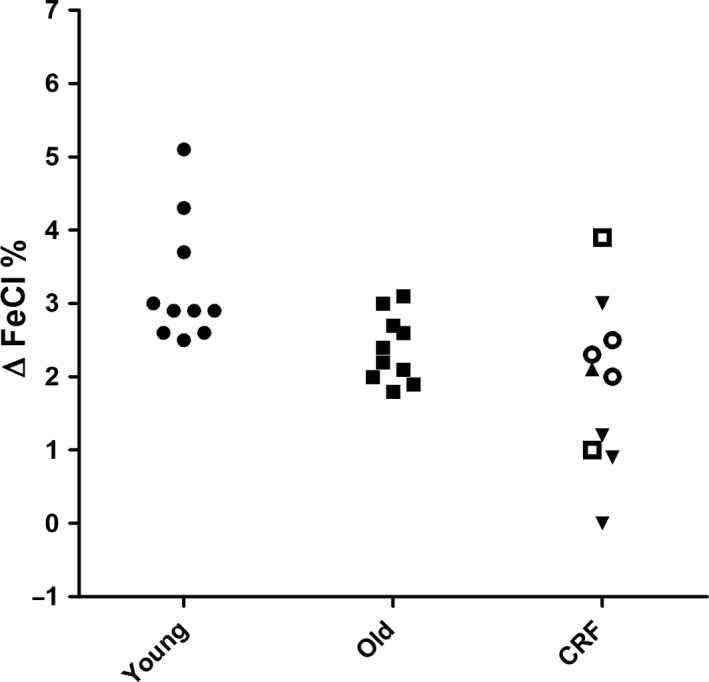
Thiazide test. Maximal increase in fractional excretion of chloride (∆FeCl) after administration of hydrochlorothiazide in young healthy individuals (young), older healthy indivduals (old), and patients with compromised renal function (eGFR 60–89 mL/min/1.73 m^2^ □, eGFR 45–60 mL/min/1.73 m^2^ ▼, eGFR 30–44 mL/min/1.73 m^2^ ○ and eGFR 15–29 mL/min/1.73 m^2^ ▲).

**Figure 3:**
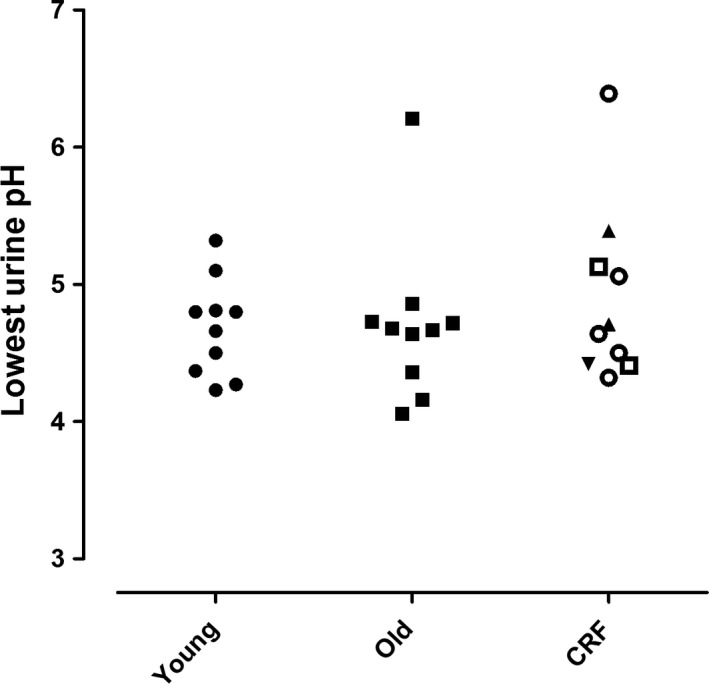
Furosemide Fludrocortisone (FF) test. Urine pH after administration of furosemide and fludrocortisone in young healthy individuals (young), older healthy individuals (old), and patients with compromised renal function (eGFR 60–89 mL/min/1.73 m^2^ □, eGFR 45–60 mL/min/1.73 m^2^ ▼, eGFR 30–44 mL/min/1.73 m^2^ ○ and eGFR 15–29 mL/min/1.73 m^2^ ▲).

**Figure 4:**
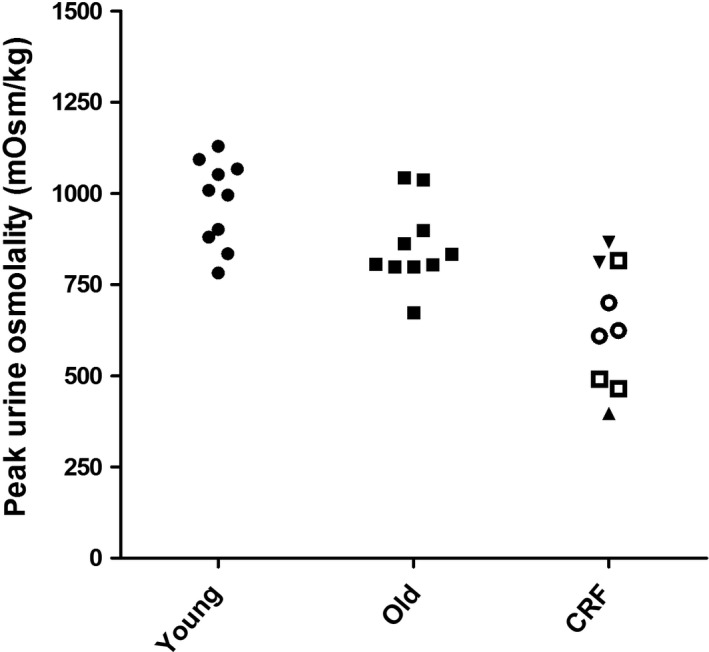
DDAVP test. Maximal urine osmolality after administration of desmopressin in young healthy volunteers (young), older healthy volunteers (old), and patients with compromised renal function (eGFR 60–89 mL/min/1.73 m^2^ □, eGFR 45–60 mL/min/1.73 m^2^ ▼, eGFR 30–44 mL/min/1.73 m^2^ ○ and eGFR 15–29 mL/min/1.73 m^2^ ▲).

We performed 129 tubular function tests in 53 individuals. No serious adverse events occurred. Test results are shown in Figures 1, 2, 3, 4 and Tables S1‐S4. Urine chloride was below the level of detection of 20 mmol/L in 24/570 samples of the furosemide and thiazide tests.

### Young healthy individuals

Clinical characteristics of young healthy individuals are given for each separate test in the supplementary Tables S1–S4. Overall, there were more female than male participants. Median age was 21 years (IQR 21‐24). Test results were in line with earlier reports. The median maximal increase in FeCl (∆FeCl) was 16.4% (range 10.3–26.3) after furosemide and 2.9% (range 2.5–5.1) after hydrochlorothiazide (Figs. 1 and 2, Tables S1 and S2). In all subjects urine pH was below 5.3 in the FF test (Fig. 3, Table S3). Median maximal urine osmolality after DDAVP was 1002 mosmol/kg H_2_O (range 782–1129) (Fig. 4, Table S4).

### Older healthy individuals

Clinical characteristics per test are given in the supplementary Tables S1–S4. Overall, there were more male than female participants. Median age was 67 years (IQR 63–68). The decrease in urine pH after FF was similar to young healthy individuals (*P* = 0.35). Older individuals showed an attenuated response to furosemide, thiazide and DDAVP compared to young healthy individuals (*P* < 0.05). In five subjects ∆FeCl remained below the lower end of the range of young healthy individuals (10.3%) after furosemide and in six subjects ∆FeCl remained below the lower end of the range of young healthy individuals (2.5%) after hydrochlorothiazide (Fig. 1 and 2, Table S1 and S2). Median maximal urine osmolality after DDAVP was 820 mosmol/kg H_2_O (range 674–1044) (Figure 4). All but one of the older subjects lowered their urine pH below 5.3 during the FF test (Fig. 3, Table S3).

### Patients with CRF

**Figure 5:**
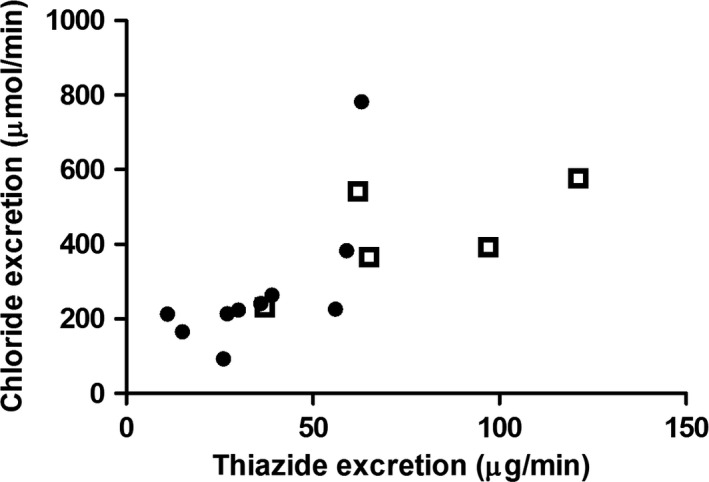
Correlation between hydrochlorothiazide excretion and chloride excretion in patients with compromised renal function (closed circles) and healthy volunteers (open squares)

Clinical characteristics per test are given in the supplementary Tables S1–S4. Overall, there were more male than female participants with CRF. Median age was 68 years (IQR 57–72), median eGFR 49 mL/min/1.73 m^2^ (IQR 33–61) and median urinary alpha‐1‐microglobulin excretion 15 mg/10 mmol creatinine (IQR 7–28). The underlying kidney disease was considered to be renovascular in nine patients, secondary focal segmental glomerulosclerosis in three patients and primarily of urologic origin in two patients. Eleven patients with CRF used an ACEi or ARB and four patients with CRF were on chronic diuretic therapy (which were stopped per the particular test protocol). Patients with CRF showed a wide variation with respect to response to furosemide and thiazide (Figs. 1 and 2). Overall the increase in FeCl after hydrochlorothiazide in CRF patients was lower compared to healthy individuals (*P* < 0.01). In contrast, the increase in FeCl after furosemide was not lower in CRF patients compared to healthy individuals (*P* = 0.12). In two subjects ∆FeCl remained below the lower end of the range of young healthy individuals (10.3%) after furosemide and in seven subjects ∆FeCl remained below the lower end of the range of young healthy individuals (2.5%) after hydrochlorothiazide. The peak of FeCl after hydrochlorothiazide occurred later in patients with CRF, although the difference was not statistically significant. In view of the wide range in ∆FeCl in response to hydrochlorothiazide, we determined hydrochlorothiazide concentrations using LCMS in urine samples collected at the time of maximal ∆FeCl. Urine hydrochlorothiazide excretion varied between patients and was not explained by variations in eGFR (data not shown). However, we observed a significant correlation between hydrochlorothiazide excretion and chloride excretion (Fig. 5). In all but one CRF patient, urine pH fell below 5.3 during the FF test. Lowest urine pH did not significantly differ between CRF patients and healthy individuals (*P* = 0.97). Patients with CRF showed an attenuated response to DDAVP compared to healthy individuals (*P* < 0.01), with a median maximal urine osmolality after DDAVP of 624 mosmol/kg/H_2_O (range 397–866) (Fig. 4).

## Discussion

Tubular function tests can be used to pin‐point a tubular defect of electrolyte transport to a specific tubular segment or function, after which other tests might detect the underlying cause, for example, genetic testing in presumed genetic renal tubular disorders. While the latter diagnostic technique seems to be taking over the role of tubular function testing in some disorders, we should realize that genetic testing is not readily available in large parts of the world and that tubular function testing can also be very instrumental and informative in disorders without (known) genetic basis. In this study we tested whether reference values for tubular function tests obtained in young healthy individuals can be extrapolated to older individuals and patients with compromised renal function.

The response to FF was similar to young healthy individuals in nearly all older individuals and patients with CRF. This is in line with earlier reports that showed that patients with CRF are still capable of lowering their urine pH (Wrong and Davies 1959; Tizianello et al. 1980). This may partly be explained by the reduced ammonium generation in patients with CRF, which limits the buffer capacity. Interestingly, one of the patients in both the older and CKD groups did not acidify their urine within 4 h. However, this could still be due to the 4 h follow‐up being too short in these particular patients and we therefore cannot exclude that these patients are still be able to (normally) acidify their urine. The responses to furosemide, hydrochlorothiazide and DDAVP, however, differed between young healthy individuals, older individuals and patients with CRF. If one would apply the often quoted reference values for the furosemide and thiazide test to elderly and patients with CRF, about 50% of subjects would have an abnormal test result. Colussi et al. (2007) previously showed that an increase in ∆FeCl in response to hydrochlorothiazide of less than 2.3% diagnoses Gitelman syndrome with a sensitivity of 93% and specificity of 100%. Using this cut‐off value, none of the healthy young adults but five out of ten older individuals and six out of ten patients with CRF, would have a false‐negative test result compatible with this diagnosis. CRF patients furthermore showed a remarkable wide variation in response. We hypothesized that this variation could be due to differences in tubular secretion. Therefore, we measured urine hydrochlorothiazide concentrations in all patients with CRF and in five healthy volunteers at the time of maximal FeCl during the thiazide test. Patients with CRF had a lower median thiazide excretion than young healthy individuals (33 *μ*g/min vs. 65 *μ*g/min, *P* = 0.01). There was a correlation between hydrochlorothiazide excretion and chloride excretion (Fig. 5, *R* ^2^ = 0.48, *P* < 0.01). The urine hydrochlorothiazide concentration did not correlate with eGFR. This indicates that the often lower and variable response to hydrochlorothiazide in patients with CRF is possibly explained by a variable tubular secretion of hydrochlorothiazide. Hydrochlorothiazide and furosemide are excreted from the blood into the proximal tubular lumen via organic anion transporters (OAT), mainly OAT1. OAT1 is inhibited by uremic toxins which could explain the reduced tubular excretion in CRF (Hsueh et al. 2016). The individual variation within the group of patients with CRF in OAT‐mediated transport can additionally be explained by polymorphisms of OAT expression, the underlying kidney disease, co‐medication and competition with other anions (Nigam et al. 2015). An important difference between the furosemide and thiazide test we performed is the adjustment of furosemide dose for eGFR. This could have led to relatively better urinary availability of furosemide compared to hydrochlorothiazide in participants with CRF, and thereby to less difference in response to furosemide than hydrochlorothiazide compared to the healthy young adults. Additional studies are needed to investigate whether tubular secretion of hydrochlorothiazide and furosemide are disturbed in elderly as well and to see if chloride excretion factored for hydrochlorothiazide or furosemide excretion is a more informative parameter. Another explanation for the variation in response encountered in CRF patients could be a difference in NCC expression. NCC expression could vary due to the underlying disease, plasma potassium and aldosterone levels and could be affected by previous drug use, albeit all possible confounding drugs were stopped beforehand (Rojas‐Vega and Gamba 2016).

In response to DDVAP, older individuals and patients with CRF had a lower maximal urine osmolality than young healthy individuals. The age‐dependent decrease in maximal urine concentrating ability is in line with the results of Tryding et al. (1988). These authors performed DDAVP tests in 212 healthy adults aged 20–80 years. Peak urine osmolality declined with age ranging from 982 ± 214 mOsm/kg at 20 years to 823 ± 278 mOsm/kg at 80 years.

While our study shows that results of renal tubular function tests are dependent on age and kidney function, the main limitation of this study is the low number of participants which precludes true definition of reference values for older individuals and patients with compromised kidney function. Besides this, it would have been informative to obtain standard urinary concentrations of furosemide and hydrochlorothiazide to give insight into the underlying mechanisms explaining the differences in test results between individual patients and groups. Another limitation is the urinary chloride concentration that was below the level of detection in a small amount of individuals in both baseline samples. We performed calculations using a urine chloride concentration of 20 mmol/L in these patients, but this could have led to incorrect higher baseline FeCl values and thereby lower ∆FeCl values in these individuals. However, when we excluded these individuals, the overall conclusion did not change. Another limitation concerns the heterogeneity of CRF patients as these patients have different underlying kidney diseases and (chronic) medication use. Chronic diuretic use could have influenced the natriuretic response according to the braking phenomenon, which is postdiuresis sodium avidity after diuretic use, despite that these drugs were stopped for 7 days prior to the test (Ellison 1999). This bias cannot fully explain the results as only one patient with chronic thiazide use underwent a thiazide test (and did not show a reduced response) and only two patients with chronic furosemide use underwent a furosemide test of which one showed a reduced response.

In conclusion, reference values for tubular function tests obtained in young healthy adults cannot be readily extrapolated to older subjects or patients with compromised renal function, except for the FF test. Larger studies with measurement of urine drug concentrations would be necessary to determine correct and clinically relevant reference values in these subject categories. For now, we should take the results of this validation study into account when interpreting results from patients of older age or with compromised renal function.

## Conflict of Interest

None declared.

## Data Accessibility

## Supporting information




**Table S1**: Furosemide test results

T**able S2:** Thiazide test results

**Table S3**: Furosemide Fludrocortisone test results

**Table S4**: DDAVP test results
