# Supplementary material for: Reference values of renal tubular function tests are dependent on age and kidney function
Source: Physiol Rep. 2017 Dec 7;5(23):e13542. doi: 10.14814/phy2.13542 (PMC5727289; doi:10.14814/phy2.13542)
Supplement: Supplementary file 1 — Table S1: Furosemide test results [file PHY2-5-e13542-s001.docx]

**Table S1**: Furosemide test results

|  | Young healthy individuals (N=10) | Older healthy individuals (N=10) | CRF patients  (N=10) |
| --- | --- | --- | --- |
| Age (years) | 22 (21-26) | 67 (62-73) * | 69 (55-73) * |
| Gender (M/F) | 2/8 | 8/2 * | 7/3 |
| Body weight – start (kg) | 65 (62-70) | 82 (72-96) * | 90 (78-109) * |
| Body weight – end (kg) | 63 (60-69) | 81 (71-95) * | 90 (77-109) * |
| Systolic BP – start (mmHg) | 124 (115-137) | 131 (120-146) | 166 (156-172) * |
| Systolic BP – end (mmHg) | 124 (115-137) | 129 (126-145) * | 159 (149-165) * |
| Diastolic BP – start (mmHg) | 72 (66-78) | 75 (71-79) | 85 (78-89) * |
| Diastolic BP – end (mmHg) | 69 (67-80) | 75 (70-81) | 82 (75-92) * |
| Pulse – start (bpm) | 70 (64-85) | 67 (59-71) | 75 (63-82) |
| Pulse – end (bpm) | 63 (59-78) | 65 (57-70) | 65 (59-78) |
| Serum creatinine – start (umol/l) | 65 (59-79) | 77 (72-85) * | 118 (102-151) * |
| Serum potassium – start (mmol/l) | 4.2 (4.1-4.3) | 4.3 (4.1-4.5) | 4.5 (4.4-4.7) * |
| Serum sodium – start (mmol/l) | 141 (140-141) | 141 (139-142) | 138 (137-140) |
| Baseline FeCl (%) | 0.99 (0.78-1.76) | 1.23 (0.88-1.56) | 1.61 (1.08-1.96) |
| Maximal FeCl (%) | 17.8 (15.9-21.5) | 12.1 (9.8-16.4) * | 15.5 (12.9-18.6) |
| Maximal ∆FeCl (%) | 16.4 (14.9-19.9) | 11.0 (8.3-15.1) * | 14.0 (11.6-16.8) |
| Time max FeCl (minutes) | 225 (210-240) | 240 (240-278) | 240 (240-308) |

Median values with interquartile ranges

M= male

F= female

BP = blood pressure

FeCl: fractional chloride excretion (%)

∆FeCl = maximal change in FeCl compared to baseline FeCl

* P<0.03 compared to young healthy individuals
