# Supplementary material for: Reference values of renal tubular function tests are dependent on age and kidney function
Source: Physiol Rep. 2017 Dec 7;5(23):e13542. doi: 10.14814/phy2.13542 (PMC5727289; doi:10.14814/phy2.13542)
Supplement: Supplementary file 2 — Table S2: Thiazide test results [file PHY2-5-e13542-s002.docx]

T**able S2:** Thiazide test results

|  | Young healthy individuals (N=10) | Older healthy individuals (N=10) | CRF patients  (N=10) |
| --- | --- | --- | --- |
| Age (years) | 21 (21-24) | 67 (62-69) * | 65 (55-72) * |
| Gender (M/F) | 3/7 | 8/2 * | 7/3 |
| Body weight – start (kg) | 65 (56-70) | 83 (74-94) * | 82 (77-109) * |
| Body weight – end (kg) | 64 (56-69) | 82 (74-94) * | 82 (77-109) * |
| Systolic BP – start (mmHg) | 130 (122-136) | 138 (130-158) | 154 (137-182) * |
| Systolic BP – end (mmHg) | 125 (115-141) | 135 (128-149) | 177 (146-180) * |
| Diastolic BP – start (mmHg) | 70 (67-84) | 80 (79-88) * | 78 (68-89) |
| Diastolic BP – end (mmHg) | 76 (69-81) | 80 (77-89) * | 84 (77-87) * |
| Pulse – start (bpm) | 79 (64-86) | 73 (61-86) | 68 (63-74) |
| Pulse – end (bpm) | 62 (45-72) | 64 (58-72) | 57 (52-66) |
| Serum creatinine – start (umol/l) | 68 (58-75) | 77 (70-86) * | 124 (112-142) * |
| Serum potassium – start (mmol/l) | 4.2 (3.9-4.2) | 4.2 (4.1-4.4) | 4.6 (4.3-4.9) * |
| Serum sodium – start (mmol/l) | 140 (138-141) | 141 (139-142) | 139 (138-142) |
| Baseline FeCl (%) | 1.08 (0.891.40) | 1.02 (0.75-1.20) | 1.61 (1.30-2.52) * |
| Maximal FeCl (%) | 4.3 (3.7-5.0) | 3.4 (3.0-3.9) * | 3.8 (2.5-5.0) |
| Maximal ∆FeCl (%) | 2.9 (2.6-3.9) | 2.3 (2.0-2.8) * | 2.1 (0.9-2.6) * |
| Time max FeCl (minutes) | 300 (270-300) | 285 (240-390) | 420 (270-510) |

Median values with interquartile ranges

M= male

F= female

BP = blood pressure

FeCl: fractional chloride excretion (%)

∆FeCl = maximal change in FeCl compared to baseline FeCl

* P<0.03 compared to young healthy individuals
