# Supplementary material for: Reference values of renal tubular function tests are dependent on age and kidney function
Source: Physiol Rep. 2017 Dec 7;5(23):e13542. doi: 10.14814/phy2.13542 (PMC5727289; doi:10.14814/phy2.13542)
Supplement: Supplementary file 3 — Table S3: Furosemide Fludrocortisone test results [file PHY2-5-e13542-s003.docx]

**Table S3**: Furosemide Fludrocortisone test results

|  | Young healthy individuals (N=10) | Older healthy individuals (N=10) | CRF patients  (N=10) |
| --- | --- | --- | --- |
| Age (years) | 21 (21-24) | 68 (74-89) * | 62 (52-72) * |
| Gender (M/F) | 4/6 | 8/2 | 6/4 |
| Body weight – start (kg) | 66 (61-71) | 80 (74-89) * | 87 (78-107) * |
| Body weight – end (kg) | 64 (59-69) | 79 (72-88) * | 85 (77-106) * |
| Systolic BP – start (mmHg) | 127 (119-135) | 142 (127-148) * | 147 (143-178) * |
| Systolic BP – end (mmHg) | 117 (108-126) | 131 (122-156) | 147 (137-169) * |
| Diastolic BP – start (mmHg) | 71 (64-82) | 74 (69-87) | 78 (69-86) |
| Diastolic BP – end (mmHg) | 69 (61-74) | 77 (73-87) * | 74 (70-86) |
| Pulse – start (bpm) | 80 (64-95) | 75 (65-88) | 63 (61-69) |
| Pulse – end (bpm) | 68 (55-83) | 67 (59-75) | 60 (53-62) |
| Serum creatinine (µmol/l) T0 | 72 (63-84) | 75 (72-81) | 129 (112-160) * |
| Urine pH T0 | 5.6 (5.4-5.8) | 5.5 (5.4-5.9) | 5.6 (5.3-6.1) |
| Urine pH T60 | 5.8 (5.4-6.4) | 5.8 (5.0-6.4) | 5.8 (5.1-6.5) |
| Urine pH T120 | 5.6 (5.2-6.6) | 5.3 (4.7-6.2) | 5.3 (5.0-6.2) |
| Urine pH T180 | 5.2 (4.6-6.0) | 5.0 (4.5-6.0) | 4.8 (4.6-5.7) |
| Urine pH T240 | 4.9 (4.7-5.1) | 4.7 (4.4-4.9) | 4.7 (4.5-5.2) |
| Lowest urine pH | 4.8 (4.5-5.1) | 4.7 (4.3-4.8) | 4.7 (4.4-5.2) |
| Time lowest urine pH (minutes) | 180 (180-240) | 240 (180-240) | 240 (225-240) |

Median values with interquartile ranges

M= male

F= female

BP = blood pressure

T0= time 0 etc

* P<0.03 compared to young healthy individuals
