# Supplementary material for: Reference values of renal tubular function tests are dependent on age and kidney function
Source: Physiol Rep. 2017 Dec 7;5(23):e13542. doi: 10.14814/phy2.13542 (PMC5727289; doi:10.14814/phy2.13542)
Supplement: Supplementary file 4 — Table S4: DDAVP test results [file PHY2-5-e13542-s004.docx]

**Table S4**: DDAVP test results

|  | Young healthy individuals (N=10) | Older healthy individuals (N=10) | CRF patients  (N=9) |
| --- | --- | --- | --- |
| Age (years) | 21 (20-23) | 66 (58-68) * | 68 (53-73) * |
| Gender (M/F) | 7/2 | 7/3 | 6/3 |
| Body weight – start (kg) | 65 (61-71) | 81 (68-100) * | 82 (77-111) * |
| Body weight – end (kg) | 64 (61-70) | 80 (68-99) * | 81 (110) * |
| Systolic BP – start (mmHg) | 132 (120-134) | 134 (120-160) | 163 (148-179) * |
| Systolic BP – end (mmHg) | 123 (105-133) | 138 (118-160) | 157 (140-168) * |
| Diastolic BP – start (mmHg) | 74 (65-84) | 81 (75-93) | 80 (75-86) |
| Diastolic BP – end (mmHg) | 65 (60-77) | 82 (70-90) * | 76 (73-83) * |
| Pulse – start (bpm) | 79 (69-93) | 76 (62-89) | 67 (60-78) |
| Pulse – end (bpm) | 64 (52-76) | 69 (55-72) | 60 (55-67) |
| Serum creatinine T0 | 72 (61-81) | 80 (67-83) | 132 (105-160) * |
| Serum sodium T0 | 141 (139-141) | 141 (140-144) | 140 (139-143) |
| Serum sodium T360 | 141 (140-142) | 141 (140-142) | 142 (140-144) |
| Urine osmol start | 898 (775-980) | 694 (498-743) * | 510 (396-590) * |
| Max urine osmol | 1002 (869-1074) | 820 (799-934) | 624 (477-814) * |
| Time max urine osmol (minutes) | 330 (300-360) | 330 (240-360) | 360 (240-360) |

Median values with interquartile ranges

M= male

F= female

BP = blood pressure

* P<0.03 compared to young healthy individuals
